# Impact of Obesity on Vaccination to SARS-CoV-2

**DOI:** 10.3389/fendo.2022.898810

**Published:** 2022-06-20

**Authors:** Michaella-Jana C. Nasr, Elizabeth Geerling, Amelia K. Pinto

**Affiliations:** Department of Molecular Microbiology and Immunology, Saint Louis University School of Medicine, St. Louis, MO, United States

**Keywords:** COVID- 19, Moderna vaccine, Pfizer, Johnson & Johnson, SARS – CoV – 2, obesity, BMI - body mass index

## Abstract

To combat the immense toll on global public health induced by severe acute respiratory syndrome coronavirus 2 (SARS-CoV-2), new vaccines were developed. While these vaccines have protected the populations who received them from severe SARS-CoV-2 infection, the effectiveness and durability of these vaccines in individuals with obesity are not fully understood. Our uncertainty of the ability of these novel vaccines to induce protective immunity in humans with obesity stems from historical data that revealed obesity-associated immune defects to influenza vaccines. This review analyzes the efficacy of SARS-CoV-2 vaccines in humans with obesity. According to the vaccine safety and efficacy information for the Pfizer, Moderna, and Johnson & Johnson formulations, these vaccines showed a similar efficacy in both individuals with and without obesity. However, clinical trials that assess BMI and central obesity showed that induced antibody titers are lower in individuals with obesity when compared to healthy weight subjects, highlighting a potential early waning of vaccine-induced antibodies linked to obesity rates. Thus, the desired protective effects of SARS-CoV-2 vaccination were potentially diminished in humans with obesity when compared to the healthy weight population, but further studies outlining functional implications of the link between obesity and lower antibody titers need to be conducted to understand the full impact of this immune phenomenon. Further, additional research must be completed to truly understand the immune responses mounted against SARS-CoV-2 in patients with obesity, and whether these responses differ from those elicited by previously studied influenza viruses.

## Introduction

In November 2019, a new, highly infectious RNA virus in the C*oronaviridae* family, termed severe acute respiratory syndrome coronavirus 2 (SARS-CoV-2), emerged. SARS-CoV-2 infections became uncontrolled worldwide and caused a widespread illness coined coronavirus-2019 disease (COVID-19) (1). SARS-CoV-2 has infected and killed millions of individuals worldwide with numbers still rising, leaving the world suspended in a pandemic state (1, 2). The severity of this viral disease and its associated negative outcomes correlate with multiple risk factors, including age (1) and presence of other diseases (3), with obesity being a major risk factor for COVID-19 and subsequent death (1–5).

As COVID-19 continues to pose a public health threat and new, more infectious variants arise, the importance of vaccinations and booster vaccines have become more apparent (6). However, based upon previous research studying the vaccine responses to Influenza A (H1N1) in populations with high obesity rates, we predict that SARS-CoV-2 vaccine responses will wane more rapidly in individuals with obesity. Thus, patients of obesity, who are at a higher risk of severe viral infection and death by COVID-19, might not have the same duration of vaccine-conferred protection as individuals without obesity (7, 8). Further compounding the importance of studying the impact of obesity on vaccine-induced immune responses, studies done over the course of 2021 after SARS-CoV-2 vaccinations became widely available to the public indicated that obesity may be linked to breakthrough infections (9–16).

## The Etiologies and Consequences of the Obesity Pandemic

The SARS-CoV-2 pandemic is not the only current pandemic; the prevalence of obesity has tripled from 1975 to 2016 worldwide (2). Almost 2 billion people worldwide are either overweight or obese, and the global prevalence of obesity in the younger population has increased by 47% between 1980 and 2013 (17). Currently in the United States, about one third of adults and 17% of children are obese or overweight (18).

Obesity is characterized by abnormal or excessive fat accumulation that causes pathophysiology, threatening overall health (19). One standard to measure obesity is body mass index (BMI), where individuals with a BMI of > 30kg/m^2^ are classified as obese, while another is by using central obesity in which waist circumference is ≥ 80 cm for women and ≥94 cm for men (16, 19). Obesity is a multifactorial disease that is commonly caused by excess dietary intake relative to energy expenditure (2, 20). The complex etiology of obesity is not limited to overeating and sedentary lifestyles (21). This disease can develop from a mixture of genetic, physiologic, psychologic, environmental, political, and social factors. Commonly used medications including corticosteroids and some anti-depressants, endocrine disruptors, lack of sleep and microbiome diversity have all been implicated in obesity (20, 22, 23). The combination of inexpensive, high caloric, fat-laden foods and decreased physical activity over the last few decades are often listed as significant contributors to the prevalence of obesity (20). In addition, the cessation of smoking may be a contributor to the obesity pandemic, as weight gain is a common consequence of smoking cessation (20, 24). As the etiological factors that lead to obesity are multifactorial and often difficult to counteract, efforts on improving treatments and vaccines for individuals with obesity are essential.

Obesity is associated with increased all-cause mortality and the risk of developing other complications, such as cardiovascular disease, hypertension, several types of cancer, diabetes mellitus, gallbladder disease, kidney disease, osteoarthritis, and stroke (2, 17). Additionally, obesity is shown to increase the risk of acquiring respiratory tract infections, like SARS-CoV-2 and influenza A virus and can impede pulmonary function through decreased expiratory reserve volume, functional capacity, and lung compliance (4, 25). In the obese state, pulmonary function is compromised due to higher abdominal fat which can also decrease diaphragm excursion (4). Treatment of obesity and its related sequelae requires approximately 21% of the United States health care expenditure, which poses an economic burden on top of a public health concern (26). Thus, the pandemic state of obesity, coupled with its related illnesses and risks, makes having appropriate medical tools to counteract rising obesity rates — the treatment, prevention, and surveillance — a public health necessity (18, 27). As such, it is imperative that scientific efforts and funding are geared toward obesity research.

## The Effects of Obesity on the Immune System

Underlying the association between poor vaccine responses and obesity is the effect of obesity on the immune system. Obesity is associated with immune cell-mediated inflammation (7, 28), and the influence of this inflammation on immune responses is only now beginning to be understood. Expansion of adipose tissue is linked to increased inflammation (29). While adipose tissue in individuals with a healthy weight predominately contains regulatory and suppressive cytokines including IL-4, IL-10, IL-13, and IL-33, adipose tissue in individuals who are obesity is associated inflammatory cytokines including IL-1β, IL-6, IL-12, IL-18, TNF-α, and IFN-γ (30–32). Adipokines and cytokines direct the immune responses to pathogens and the presence or absence of adipokines and cytokines during vaccination can alter adaptive immune response development leading to differences in the protective efficacy following vaccination.

Previous studies have also reported an association between leptin and altered influenza vaccine responses (33, 34). Leptin, an adipokine (cell-signaling hormone) that regulates appetite and controls energy metabolism (35), is found throughout the immune system on innate immune cells (macrophages, dendritic cells, and mast cells) and on adaptive immune cells (B and T cells). Leptin plays a major role in the chronic inflammation found in patients with obesity (35). Leptin’s effect on neutrophils is especially important in COVID-19 progression in the obese state: increased leptin levels, characterized by increased neutrophilic lung inflammation, causes more severe lung injury (1). Higher leptin in obesity also correlates with a decreased levels of T_reg_ cells, resulting in more pro-inflammatory than anti-inflammatory cytokine expression, and an increased activation of neutrophils (1). Leptin has also been shown to direct T cell proliferation and reactivity by activating the JAK/STAT pathway, thereby enhancing immune inflammation (36, 37). However, based on some studies in humans and animals, it is thought that the hyperleptinemia state in patients with obesity may eventually induce leptin resistance (35, 38, 39). Leptin sensitivity and resistance remain active areas of research and the clinical criteria for defining leptin resistance and its diagnostic use have not yet been established.

As previously described, the expansion of adipose tissue in the obese state is known to contribute to chronic, low-grade inflammation due to adipocyte hypoxia and resulting immune cell infiltration (21, 40). Chronic inflammation not only directly increases the risk for cardiovascular disease (CVD) and diabetes, but also causes upregulation of proteins and cytokines (like p38 MAPK and TNF-α), which can cause tissue damage (1, 21, 40) and result in a positive feedback loop that further promotes inflammation. This chronic immune stimulation can weaken the humoral responses and cell-mediated immunity, specifically lowering T cell response magnitude and increasing the time it takes for such responses to be mounted (7, 8). For example, individuals with obesity show suppressed T cell activation and differentiation in response to influenza infection when compared to individuals of healthy weights. Also, decreases in T cell production of effector molecules like IFN-γ and granzyme B is associated with obesity (7, 28). Further, obesity can induce B cell defects, including a lower frequency of regulatory B cells (with phenotypes CD19^+^ CD27^+^ CD38^High^, CD19^+^ CD24^High^ CD38^High^, and CD19^+^ CD24^High^ CD38^High^ IL-10^+^), in response to infection (41, 42). This obesity-induced immune suppression, particularly regarding B cell impairment, makes it unsurprising that individuals with obesity have inadequate seroconversion rates following vaccination. This is shown with decreased antibody titers in response to vaccination to hepatitis B, tetanus, and rabies (43). In addition to decreased antibody titers following vaccination, antibody responses also wane rapidly in individuals with obesity compared to people of healthy weights (44). Furthermore, the influenza vaccine is not as effective in individuals with obesity compared to individuals of healthy weights, potentially in part due to decreased antibody titers, decreased CD8+ T-cell activation, and decreased production of functional proteins IFN-ɣ and granzyme B (44, 45).

Data gathered from previous vaccine trials have shown that development of personalized vaccines might be necessary to surmount the immune suppression induced by obesity (7). Identifying the effects of obesity on immune responses mounted post-influenza infection or vaccination have provided key insights on how to improve vaccine design so vaccination can better protect populations with high obesity rates from severe viral disease and confer lifelong protection that does not rapidly wane.

## Effects of Obesity on SARS-CoV-2 Infection and Severity

First identified during the 2009 influenza A (H1N1) pandemic, obesity is a major risk factor for severe respiratory viral infection and increased mortality of infected individuals (19, 46). Obesity is associated with increased hospitalizations, intensive care unit (ICU) admissions, intubations, invasive mechanical ventilations (IMV), and viral exposures when compared to patients of healthy weight (19, 47–49). The exacerbation of COVID-19 disease progression in the population with obesity is thought to be linked to higher viral load and slower antiviral responses seen in COVID-19 patients with obesity (19).

Similar to the H1N1 pandemic, enhanced viral disease severity among COVID-19 patients with obesity have been noted amidst the SARS-CoV-2 pandemic. First, hospital admissions, stays, and recovery time of COVID-19 patients with obesity are longer than those of individuals of healthy weights (19, 50). Specifically, patients with obesity took 19 ± 8 days to achieve a negative nasopharyngeal swab for SARS-CoV-2 resolution compared to individuals of healthy weights who took 13 ± 7 days (50). Furthermore, among ICU COVID-19 patients, a higher BMI was reported in comparison with non-ICU patients (19). Interestingly, in a study conducted in Italy among 1591 ICU patients, 68% had at least one comorbidity, including hypertension, CVD, and diabetes, which are all obesity-related illnesses (51). Similarly, IMV indications were positively correlated with elevated BMI, and were greatest in COVID patients with a BMI ≥ 35 kg/m^2^ (19). Obesity also increased the risk of pneumonia in COVID-19 patients compared to individuals without obesity (52). In addition, acute respiratory distress syndrome (ARDS), embodied by respiratory failure and hypoxemia, is a severe consequence of COVID-19, and reports highlight that obesity increases the ARDS risk and incidence in COVID-19 patients compared to COVID-19 patients of healthy weights (19). Due to the severe effects of COVID-19 infections in individuals with obesity and their diminished immune responses contributing to viral disease progression, targeted treatments are necessary to avoid long-term health effects and death in this patient population. Similar to data from influenza infections, SARS-CoV-2 patient outcome statistics again highlight the importance of formulating personalized vaccines or modifying vaccine schedules on a per patient basis.

## SARS-CoV-2 Vaccine Trials and Obesity

To combat the SARS-CoV-2 pandemic, multiple vaccine platforms were adapted for rapid Phase I, II and III clinical trials with limited or emergency use. Four vaccines are currently approved for use and being used worldwide from the following manufacturers: Moderna, Johnson & Johnson (J&J, Jansen Ad26), AstraZeneca and Pfizer-BioNTech (Pfizer, BNT162b2 mRNA). Along with the Pfizer-BioNTech vaccine, the Moderna vaccine was granted full FDA approval. During the clinical trial phases, Pfizer, Moderna, and J&J collected data on vaccine efficacy (VE) in individuals with obesity against the ancestral SARS-CoV-2 strain and alpha variant of concern; however, AstraZeneca has not provided data about VE in subjects with obesity. Thus, the remainder of our review will focus on the three vaccine platforms currently used in the United States that assessed VE in the context of obesity: Moderna, J&J and Pfizer.

### Pfizer BioNTech BNT162b2

The Pfizer BNT162b2 formulation is an mRNA vaccine encoding the full-length spike (S) protein (53, 54). The phase III trial for this vaccine included a sample size of about 43,000 people and was a randomized, placebo-controlled trial (53). In this trial, participants received 2 doses (21 days apart) of either the vaccine or a placebo (53). Independent of weight, the VE was 95% in people without a previous COVID-19 infection who received the vaccine compared to the ones who received the placebo (53).

In this phase III trial, 13,218 subjects were classified as having obesity based on a BMI ≥ 30 kg/m^2^ (53). The vaccinated group had 6,556 participants with obesity, and the placebo group had 6,662 participants with obesity (53). Based on results from this trial, obesity did not impact VE (53). Specifically, this study looked seven days after the second dose where VE was 95.4% in individuals with obesity versus 94.8% VE in subjects without obesity (53). When these data were stratified for age, no significant differences in VE were noted; VE in younger adults with obesity (ages:16-64) was ~95%, whereas in older adults with obesity (age >65), VE was 100% (53). While these data are promising, VE tests were completed only seven days after the second dose; VE studies were not conducted at later time points post-vaccination. Similarities in the vaccine responses early after vaccination between either individuals with obesity or of healthy weights have previously been seen in studies of the seasonal influenza vaccine (44). However, in such studies, virus specific antibody responses wane significantly in individuals with obesity after one year as compared to antibody titers of individuals of healthy weights (44).

The impact of obesity on the durability of vaccine-conferred protection is critical to understand as multiple studies have shown that a booster or third dose of a SARS-CoV-2 vaccine helps provide protection as immunity against this virus wanes (55–58). In at least one limited study consisting of 1,060 subjects, antibody levels to SARS-CoV-2 following the Pfizer BNT162b2 mRNA vaccination were measured at baseline, 21 days post first dose, 30-40 days post second dose and 90-100 days post second dose and compared between subjects with obesity versus those without. Consistent with studies conducted following seasonal influenza virus vaccination, early antibody titers were essentially equivalent between individuals with obesity versus those without, but by one month post second dose, antibody titers reported for subjects with obesity were significantly lower than those noted for subjects of healthy weight. Similarly, antibody titers of individuals with obesity were further significantly reduced at three months post-second dose when compared to levels reported for individuals of healthy weights (59). However, these studies only report a waning of the antibody response and did not address the implications of decreased antibody titers in individuals with obesity. Further studies using functional assays need to be conducted to determine if lower antibody titers correlate with functional defects in the ability for individuals with obesity.

In concordance with the previously described study, a study done by Watanabe et al. enrolled 22 adult subjects experiencing central obesity and at least one obesity-associated comorbidity, such as hypertension. This study was conducted to examine the impact of obesity on immune responses elicited by the Pfizer BNT162b2 mRNA vaccine (60). Prior to entering the study, these subjects were not vaccinated against SARS-CoV-2 and upon enrollment into the study, patients were placed on dietary intervention, with energy requirements calculated by adjusting for the physical activity level of each individual (60). Patients were vaccinated against SARS-CoV-2, and data generated following both doses in the vaccine schedule highlighted that BMI was inversely correlated with both cell-mediated and humoral immune responses (60). Interestingly, while following the dietary restrictions established upon enrollment into this study, patients lost ~10% of their body weight (accompanied by metabolic improvements), and this weight loss positively correlated with improved cell-mediated responses following vaccination (60). This study provides a unique perspective on vaccination in individuals plagued by obesity as it highlights that losing weight, or improving metabolic health, may counteract the immune defects that occur during priming in the obese state, showing that these cellular changes can be reversed.

### Moderna mRNA-1273

The Moderna SARS-CoV-2 vaccine also utilizes an mRNA platform, coined mRNA-1273. This vaccine encodes a stabilized pre-fusion form of the S protein, a desirable vaccine design due to stabilized pre-fusion viral glycoproteins being highly immunogenic (54, 61). A randomized, placebo-controlled trial was conducted with a sample size of 30,351 participants (61). These participants were adults (age >18). Similar to the Pfizer trial, two doses were given to participants, but they were administered 28 days apart instead of 21 (61). The overall efficacy of this vaccine was 94.1%, but when VE was measured separately only among the vaccinated individuals with obesity (901 subjects), VE only dropped slightly from 94.1% to 91.2%. However, 11 cases of COVID-19 were reported in the vaccinated group, one of which was severe and did occur in a subject with obesity (61). On the other hand, 185 COVID-19 cases were reported in the placebo group. Of the 185 cases, 30 cases were severe, and one case led to death (61). Similarly, these data were further broken down to examine infection rates about placebo subjects with obesity. Of the 30 severe COVID-19 cases reported among the placebo group, 11 of them were diagnosed in subjects with obesity. Thus, overall, mRNA-1273 VE appeared to be similar between subjects with obesity or those of healthy weights, although reported viral disease severity did trend higher in the subjects with obesity.

However, similar to the Pfizer BioNTech BNT162b2 phase three trial, the primary endpoint for this study was 15 days after the second vaccine dose was given. While the VE reported in this clinical trial appears promising for individuals with obesity, as noted above, prior research suggests that it is essential to look at the long-term durability of VE to conclude if obesity impacts SARS-CoV-2 vaccine-conferred protection. Further studies should be conducted to examine the durability of vaccine specific responses in humans with obesity to determine if administering booster vaccinations earlier might could sustain long-term immunity to viral pathogens.

### Janssen/Johnson & Johnson Ad26.CoV2.S

J&J developed a replication-incompetent adenovirus serotype 26 vectored vaccine (Ad26.CoV2.S) and similarly conducted a randomized, double blind, placebo-controlled trial. The sample size of this trial was 39,321 individuals (62). In this vaccine schedule, only one dose is administered to each subject. Fourteen days post-vaccination, VE was reported as 67.4%. By 28 days post-vaccination, VE was noted to be 66.2%.

In this trial, 28.5% of the cohort were classified as having obesity based on BMI > 30kg/m^2^. VE in this group was 66.8% 2 weeks post-vaccination and 65.9% 28 days post-vaccination. No deaths were reported among subjects with obesity in the vaccinated group, but 6 out of 7 fatalities in the placebo group were subjects with obesity (62). Based on this phase three trial, the reported VE was consistent in individuals with obesity compared to subjects of healthy weights as reported for the Pfizer BioNTech and Moderna formulations, but viral disease severity did trend higher in the unvaccinated subjects with obesity. Again, although VE reported in this trial appears equivalent among subjects with obesity versus those without, tracking vaccine-specific immune responses as time progresses post-vaccination could illuminate implications for obesity on durability of VE.

## Current Research May Illustrate Effects of Obesity on Vaccination Against SARS-CoV-2

Although information provided from the SARS-CoV-2 vaccine trials did not appear to show differences in terms of VE between individuals with obesity versus those of healthy weights, several longitudinal studies have highlighted some immune defects in populations with high obesity rates. One longitudinal study measured the effects of central obesity on Pfizer/BioNTech vaccination in 86 healthcare workers in Italy (16). This study showed that central obesity was associated with lower antibody titers following vaccination, but this phenomenon occurred independently from BMI (16). In a study looking at the antibody titers of individuals who were overweight, obese or of healthy weight between the first and second dose of the Pfizer/BioNTech vaccine, Pellini et al. noted that a single vaccination activated the humoral immune response in individuals of healthy weights, but some subjects with obesity or who were reported as being overweight (age >47 and BMI >25 kg/m^2^) did not have a change in their IgG antibody levels (41). The authors concluded that IgG antibody titers in populations classified as having healthy weights or being young in age were higher than antibody responses in populations classified as being overweight or older in age, but more research has to be done regarding its direct correlation to protection against severe viral disease (41). While both studies noted a difference in antibody responses following vaccination, the results differ as to the association with BMI and central obesity. The current randomized, controlled trials assessed different measures of obesity, but both showed that obesity can be associated with a lower antibody titer following vaccination to COVID-19. These studies suggest that variabilities in how vaccine efficacy is measured, the time elapsed post vaccination, and the specific vaccine formulation administered can lead to confounding results. However, these studies, as well as the phase three trial discussed above, reveal that vaccination of the individuals with obesity against SARS-CoV-2 is effective, at least for conferring short-term protection. While the duration of the effectiveness may be shortened, there is a window of time where protection is observed. As the SARS-CoV-2 pandemic is ongoing, follow up studies comparing vaccine-induced immune responses among individuals with obesity versus those of healthy weights are essential studies to conduct.

## Discussion

Data from the phase III SARS-CoV-2 vaccine trials and subsequent clinical trials conducted to measure immune responses primed in the obese state after inoculation produced results that contradict each other, likely due to the physiological complexity of obesity. Overall, the vaccine trials of the three SARS-CoV-2 vaccines administered in the US show that they are efficacious in individuals with obesity; however, statistical analyses were not completed on these data to validate the outcomes. Moreover, other clinical trial cohorts report decreased antibody titers and weakened immune responses following SARS-CoV-2 vaccination in individuals who are overweight or obese. The current research is limited, and the contradiction sparks a need for further studies to be conducted. The clinical trials that measured central obesity did not find an association to BMI and a decreased immune response. This finding does not match with Pellini et al. (41) and their study on BMI, in which a high BMI was associated with lower antibody titers. Watanabe et al. explain that BMI is not an appropriate way of measuring obesity, highlighting that central obesity presents a more accurate measure of the severity of obesity and its sequalae in those with high waist circumferences, a phenomenon that has shifted the field of obesity to now focus on classifying individuals as having metabolic syndrome. Thus, different measures of obesity might indicate a varying response than what is seen in the vaccine trials, as the vaccine trials used BMI to assess efficacy of the vaccine in individuals with obesity, and it is possible that a different measure of obesity might yield a different result. Hence, more research is necessary to resolve these scientific questions.

Additionally, the study methods differed in a way that might lead to significant differences in vaccine efficacy results, such as measuring antibody titers at varying time points or using different antibody measuring kits. Moreover, the sample sizes in the clinical trials measuring the effects of obesity on vaccine responses in individuals with obesity were small compared to samples sizes utilized in vaccine trials and not entirely representative of the general population of individuals with obesity. For example, Watanabe et al. and Pellini et al. (41, 60) recruited study participants who were healthcare workers, a group that generally has more access to health care to help with any current or future obesity-related illnesses, in contrast to the general population which may not have immediate or affordable health care access. Further, although some pre-existing medical conditions were noted for the subjects analyzed in these studies, information regarding their medication use to treat such conditions was not disclosed, potentially skewing the data generated.

Even with the present research presented, many questions remain unanswered, such as how immune responses change depending on the age demographic. The clinical trials discussed throughout this review focused on adults, but with an increasing population of children becoming overweight and obese, studies must be expanded to include subjects of varying ages to determine whether vaccines are efficacious in conferring protection against severe viral diseases in children with obesity. It is also difficult to assess how people with obesity-related comorbidities benefit from the current vaccines, especially regarding lifelong, durable protection. Furthermore, if vaccine efficacy is similar in individuals with obesity to those individuals of healthy weights during a close time frame to when the vaccine is administered, how durable vaccine efficacy is in individuals with obesity is unknown and could pose threats to public health as time post vaccination increases. It is possible that additional vaccine boosters will be necessary for individuals with obesity to achieve protective and durable vaccine-induced immunity.

## Conclusion

We believe that more research needs to be done to assess the impact of obesity on vaccination to SARS-CoV-2. The currently reported research is contradictory, and numerous questions remain unanswered. We believe more work needs to be done to assess long-term efficacy of the available vaccines to SARS-CoV-2, what the best scheduling for boosters is, and how different patterns of fat distribution could be affecting immune responses to vaccination. However, it is clear that obesity hinders immune responses to vaccines and infections. As obesity rates are projected to continually rise globally, it is important to gear medical treatments towards populations with high obesity incidences and to increase awareness about non-dietary causes of obesity. Furthermore, more data needs to be gathered concerning the growing number of young individuals with obesity and if they will be protected from severe viral disease by SARS-CoV-2 vaccines. With these future research efforts in mind, we are confident that vaccine development can improve to induce long-lasting, protective immune responses in patients with obesity.

## Author Contributions

All authors listed have made a substantial, direct, and intellectual contribution to the work, and approved it for publication.

## Funding

This work was supported in part by the National Institutes of Health, National Institute of Allergy and Infectious Diseases (NIAID): 5R01AI137424-03, and in part by the Department of Defense (award no. PR192269). The funders had no role in decision to publish, or preparation of the manuscript.

## Conflict of Interest

The authors declare that the research was conducted in the absence of any commercial or financial relationships that could be construed as a potential conflict of interest.

## Publisher’s Note

All claims expressed in this article are solely those of the authors and do not necessarily represent those of their affiliated organizations, or those of the publisher, the editors and the reviewers. Any product that may be evaluated in this article, or claim that may be made by its manufacturer, is not guaranteed or endorsed by the publisher.
